# Homeless

**Published:** 1888-11-10

**Authors:** E. Henderson

**Affiliations:** Secretary to the Metropolitan Association for Befriending Young Servants


### Editor's Letter-Box.

### HOMELESS.

Will anyone give a home and clothing to a poor girl, aged 20, who is nearly blind? She is very respectable, and can scrub, take care of children, etc. If she were quite blind we could send her to an asylum ; as it is, if no one will take her she must go to the workhouse. E. Henderson,
Secretary to the Metropolitan Association for Befriending Young Servants.

